# Noncompetitive inhibition of human CYP2C9 in vitro by a commercial *Rhodiola rosea* product

**DOI:** 10.1002/prp2.324

**Published:** 2017-06-05

**Authors:** Ole Kristian Forstrønen Thu, Olav Spigset, Bent Hellum

**Affiliations:** ^1^ Department of Cancer Research and Molecular Medicine Faculty of Medicine and Health Sciences Norwegian University of Science and Technology (NTNU) Trondheim Norway; ^2^ Department of Clinical Pharmacology St. Olav University Hospital Trondheim Norway; ^3^ Department of Laboratory Medicine Children's and Women's Health Faculty of Medicine and Health Sciences Norwegian University of Science and Technology (NTNU) Trondheim Norway

**Keywords:** Commercial, CYP enzymes, CYP2C9, enzyme inhibition, ethanol inhibition, Lineweaver‐Burk, noncompetitive, *Rhodiola rosea*, supersomes

## Abstract

A commercial *Rhodiola rosea* (*R. rosea*) product has previously demonstrated CYP2C9 inhibition in humans. The purpose of this study was to provide in vitro inhibitory data for this particular interaction and to classify the mechanism of the interaction. Another aim was to examine the in vitro influence of ethanol on the CYP2C9 activity. Human CYP2C9 (wild type) isolated from a baculovirus‐infected cell system was incubated with 0.8 *μ*mol/L losartan for 20 min. Sulfaphenazole was used as a positive control. The commercial *R. rosea* product “Arctic Root” was used as test inhibitor. Formation of the CYP2C9‐produced losartan metabolite EXP‐3174 was determined by validated LC‐MS/MS methodology. Possible mechanism‐based (irreversible) inhibition was evaluated using time‐ and NADPH‐dependent inhibition assays. Kinetic constants (*K*_m_, *V*_max_, and *K*_i_) were calculated from a Lineweaver‐Burk plot. Mode of inhibition was determined. CYP2C9 was inhibited by “Arctic Root” with an IC_50_ (extract concentration yielding 50% reduction in enzyme activity) of 19.2 ± 2.7 *μ*g/mL. Inhibitor concentrations of 20 *μ*g/mL and 40 *μ*g/mL yielded K_i_ values of 16.37 *μ*g/mL and 5.59 *μ*g/mL, respectively. The Lineweaver‐Burk plot showed noncompetitive inhibition mode. No time‐ or NADPH‐dependent inhibition was observed. The presence of ethanol inhibited CYP2C9 activity in a concentration‐dependent manner. In conclusion, the commercial *R. rosea* product “Arctic Root” demonstrated noncompetitive inhibition of CYP2C9 in vitro. Further work identifying the constituents responsible for this inhibition is needed.

**Abbreviations**
AUC: area under the curve
IS: internal standard

## Introduction

*Rhodiola rosea* L. (Crassulaceae) is a plant in the Crassulaceae family, and is mainly found in arctic and mountainous habitats (Ming et al. 2005). Historically, *R. rosea* has been used for numerous medical problems, for example, headache, hernias, and kidney stones (Panossian et al. 2010). Today, the products are mainly marketed to alleviate mild depression and to increase energy levels, libido, and mental performance. The commercial *R. rosea* industry is large, with over 46 registered international companies, giving the herb a global distribution (Galambosi 2005; Edwards et al. 2012).

CYP2C9 is a part of the CYP2C family of enzymes, which accounts for about 20% of all P450 enzymes in the human liver (Niwa and Yamazaki 2012). CYP2C9 metabolizes more than 20% of all therapeutic drugs, including drugs with a narrow therapeutic index, such as phenytoin and warfarin (Miners and Birkett 1998; Danielson 2002; Niwa and Yamazaki 2012). Inhibition of CYP2C9 activity has been shown clinically relevant for numerous drug inhibitors like amiodarone, trimethoprim, and sulfaphenazole (Miners and Birkett 1998). Inhibition studies by these drugs, and also by herbal supplement extracts, have shown that CYP2C9 can be subject to different types of inhibition, including competitive inhibition (St. John's wort constituents), mixed‐model inhibition (Phikud navakot extract), noncompetitive inhibition (Bacopa monnieri extract), and mechanism‐based (irreversible) inhibition (Lindera aggregate constituent) (Miners and Birkett 1998; Obach 2000; Ramasamy et al. 2014; Wang et al. 2015; Chiangsom et al. 2016).

Several studies have evaluated the inhibitory potential of *R. rosea* toward cytochrome P‐450 (CYP) enzymes. A previously published study from our group found an in vitro inhibition of CYP3A4 enzyme activity by extracts of six *R. rosea* clones, with IC_50_ values (extract concentration yielding 50% reduction in enzyme activity) ranging from 1.7 to 3.1 *μ*g/mL (Hellum et al. 2010). We have also evaluated the in vitro inhibition potential of several commercial *R. rosea* products on CYP1A2, CYP2D6, and CYP3A4 activities, which produced IC_50_ values ranging from 7.2 to 186 *μ*g/mL (Thu et al. 2016a). The in vivo interaction potential of *R. rosea* has also been studied by some groups. Panossian et al. (2009) evaluated the influence of 50 mg/kg *R. rosea* extracts on CYP2C9 metabolism of warfarin in rats. They found a 34% increase in warfarin *C*_max_, but did not notice any significant changes in area under the curve (AUC) or anticoagulant activity. This result was opposed by Spanakis et al. (2013), who found an almost twofold increase in AUC of the CYP2C9 substrate losartan after a 50 mg/kg concurrent administration of a commercial *R. rosea* extract to six rabbits in a two‐way crossover study, concluding that *R. rosea* could be categorized as a moderate in vivo inhibitor and that a study in humans was needed. Recently, our group published a study evaluating CYP1A2, CYP2C9, CYP2C19, CYP2D6, and CYP3A4 interaction in man by a commercially available *R. rosea* product similar to that used by Spanakis et al (2013). In this study, using a two‐phase, randomized cross‐over cocktail study in 13 males, we found a 21% reduction in the EXP‐3174/losartan ratio, indicating a significant inhibition of CYP2C9 enzyme activity (Thu et al. 2016b).

For herbal products with identified bioactive constituents, in vitro studies are usually performed with isolated constituents only, yielding specific data, which can be extrapolated to herbal products with similar constituent concentration. For *R. rosea* the rosavins (rosarin, rosin, and rosavin), salidroside, and tyrosol have been suggested as bioactive constituents (Hellum et al. 2010), and the rosavins are now used as identification markers for the content of *R. rosea* in commercial products (Brown et al. 2002; Ma et al. 2011; Mudge et al. 2013). However, the constituent(s) responsible for the in vitro and in vivo CYP inhibition by *R. rosea* remains to be identified and the concentration of these constituents could not be linked to in vitro enzyme inhibition of CYP1A2, CYP2D6, or CYP3A4 in a previous experiment (Thu et al. 2016a). Consequently, it is of interest to study full‐extract solutions of *R. rosea,* as a full extract provides a more accurate depiction of what the consumers are exposed to when they ingest these products.

The inhibition of CYP enzymes can broadly be divided into two types; reversible and irreversible. By observing the enzyme kinetics of CYP metabolite production, reversible inhibitors can be further classified into subgroups: competitive, noncompetitive, or uncompetitive inhibitors (Zhang and Wong 2005). Irreversible inhibitors, or mechanism‐based inhibitors, are inhibitors which mainly satisfies four criteria (1) Time‐dependent inactivation; (2) Inactivation that is practically irreversible when removing the inhibitor; (3) The inhibitor should be converted into a reactive intermediate; and (4) The rate of inactivation typically follows Michaelis‐Menten kinetics (Zhang and Wong 2005).

Given the previous studies, where “Arctic Root” was found to be the most potent in vitro inhibitor among a selection of commercially available *R. rosea* products and also was displaying CYP2C9 inhibition in humans, this study was undertaken with the aim to provide CYP2C9 in vitro inhibition data, including the classification of the type of inhibition, using this particular *R. rosea* product.

## Materials and Methods

Losartan potassium (Sigma 61188, lot no. 0001417819), sulfaphenazole (Sigma S‐0758, lot no. 054K0975), and caffeine (Sigma C0750, lot no. 0001400932) were obtained from Sigma‐Aldrich (St. Louis, MO). EXP‐3174 (lot no. 200013283) was kindly donated from Merck (Darmstadt, Germany). Baculovirus expressed human wild‐type CYP2C9*1 (cat. no. 456258, lot no. 11293), NADPH regenerating system Solution A (31 mmol/L NADP+, 66 mmol/L glucose‐6‐phosphate, 66 mmol/L MgCl_2_ in H2O, cat. no. 451220, lot no 57465), and Solution B (40 U/mmol/L glucose‐6‐phosphate dehydrogenase in 5 mmol/L sodium citrate, cat. no. 451200, lot no 56568) were purchased from BD Biosciences (Woburn, MA). All other chemicals were of HPLC grade.

The commercial *R. rosea* product “Arctic Root” (batch/lot no. 60419, produced by Swedish Herbal Institute, Vallberga, Sweden) was obtained from a store for herbal drugs.

One “Arctic Root” tablet was weighed, grounded in a mortar, and dissolved in 15 mL 50% ethanol. Herbal constituents were extracted at 37°C for 1 h. The extraction solution was transferred to a falcon tube, centrifuged at 1562 xg for 10 min, and decanted into a new container. The residue was added 5 mL of 50% ethanol and the extraction process was repeated. The first and second extracts were pooled and the extract solution was evaporated to dryness at 40°C under a gentle stream of air overnight and weighed. Dried extracts were kept at 4°C, avoiding light. Before experiments, the extract was dissolved in a small amount of 50% ethanol to make herbal stock solutions with known concentrations. Shelf life was set to 2 weeks.

The in vitro herbal concentration range tested in the IC_50_ experiment (0–100 *μ*g/mL) was expected to cover (and exceed) the herbal concentrations occurring in the small intestine, liver, and blood in vivo. Our estimates (Hellum and Nilsen 2007) are based on the total recommended daily intake as stated by the manufacturer.

For mechanism‐based experiments, an inhibitor concentration of 20 *μ*g/mL was used, and for the enzyme kinetics experiment, inhibitor concentrations of 20 and 40 *μ*g/mL were used.

The CYP2C9*1 enzyme preparation used was a recombinant cDNA‐expressed wild‐type CYP2C9*1 prepared from a baculovirus‐infected insect cell system. CYP2C9*1 content was 278 pmol/mg protein.

CYP2C9 (12.5 nmol/L) was incubated in conical glass tubes in a shaking water bath for 15 min at 37°C in a 0.1 mmol/L potassium‐phosphate buffer (pH 7.4) containing losartan (0.8 *μ*mol/L) and a NADPH regenerating system (1.25 mmol/L NADP^+^, 3.3 mmol/L Glucose‐6‐phosphate, 3.3 mmol/L MgCl_2_, and 0.4 U/mL glucose‐6‐phosphate dehydrogenase). *R. rosea*, the positive control inhibitor sulfaphenazole (1.0 *μ*mol/L) or buffer/ethanol was added in volumes of 100 *μ*L. As the herbal solution contained ethanol, all incubations were performed in 0.8% ethanol with adequate controls. The total incubation volume was 400 *μ*L. After a 5 min acclimatizing in the water bath, the reaction was initiated by adding 20 *μ*L of the regenerating NADPH system. The reaction was terminated on ice by the addition of 200 *μ*L stop solution (acetonitrile containing the internal standard (IS) caffeine). The formation of EXP‐3174 was linear from 5 to 20 min with CYP2C9 concentrations up to at least 30 nmol/L and a losartan concentration ranging from 0.05 to 40 *μ*mol/L.

For evaluation of mechanism‐based inhibition, time‐ and NADPH‐dependent inhibition assays were applied. In the time‐dependent assay, CYP2C9 was preincubated at 37°C with *R. rosea* in the presence of the NADPH regenerating system, but without substrate. Preincubation was continued for different periods of time (0, 15, 30, and 45 min), whereafter the substrate was added to a concentration of 0.8 *μ*mol/L and the incubation was continued for another 15 min. The reaction was terminated on ice by adding 200 *μ*L of stop solution.

The NADPH‐dependent assay was performed by preincubating CYP2C9 with *R. rosea* as described above, but in the presence and absence of the NADPH regenerating system for 0 and 45 min. The reaction was initiated by adding substrate and continued as described above.

EXP‐3174 was analyzed by previously published validated LC‐MSMS methodology, with minor adjustments (Thu et al. 2016b). Details are given in the supplementary material. A standard curve of EXP3174 was constructed from 5.0 to 90 nmol/L (7 nonzero concentrations). Limit of quantitation was 5.0 nmol/L. Within‐ and between‐day coefficients of variation were <14%. The concentration of the metabolite was estimated from area ratios (metabolite/IS). Data collection and analysis was handled by Analyst Software 1.5.1 (Applied Biosystems, Waltham, MA).

The CYP2C9 activity was determined from the formation rate of EXP‐3174 from losartan when based on a total CYP2C9 amount of 5 pmol in the incubation solution and an incubation time of 15 min. Enzyme activity was expressed as pmol EXP‐3174 formed per pmol CYP2C9 and min.

The IC_50_ value of “Arctic Root” was estimated from nonlinear regression of the inhibition plot where CYP2C9 activity was plotted against increasing herbal concentrations using Sigmaplot (Sigmaplot, Ver. 13.0: Systat Software, Inc. San Jose, CA).

*K*_m_ and *V*_max_ values for CYP2C9‐mediated metabolism of losartan were estimated by incubating CYP2C9 with increasing substrate concentrations (0.4–8.0 *μ*mol/L), *K*_m_ (app) and *V*_max_ (app) were obtained in the presence of control inhibitor and *R. rosea*. Data were transformed, plotted in a Lineweaver‐Burk plot using Sigmaplot, and the mode of inhibition was determined by analyzing the plot and applying the equations presented in Table 1 (Hellum and Nilsen 2007). *K*_i_ was calculated by using the equations given in Table 1.

**Table 1:** Equations used for the calculation of *K*_i_ (Hellum and Nilsen 2007)

Type of inhibition	*K*_m_ (app)^a^	*V*_max_ (app)^a^
None	*K*_m_ b	*V*_max_ b
Competitive	*K*_m_ (1 + [I]/*K*_i_)	*V*_max_
Noncompetitive	*K*_m_	*V*_max_/(1 + [I]/*K*_i_)
Uncompetitive	*K*_m_/(1 + [I]/*K*_i_)	*V*_max_/(1 + [I]/*K*_i_)

a *K*_m_ (app) and *V*_max_ (app) are apparent *K*_m_ and *V*_max_ in the presence of inhibitors.

b *K*_m_ and *V*_max_ in the absence of inhibitor.

Data are presented as means ± SD of *n* = 3 replicates. A two‐sample *t*‐test was used to evaluate the effect of the herbal preparation and inhibitor control on CYP2C9 enzyme activity and linear regression analyses were performed on standard curves and inhibition plots. Dixon's *Q*‐test was used to identify and remove one potential outlier per dataset, if applicable (Dean and Dixon 1951). Statistical analyses were performed on SPSS (SPSS for Windows, Rel. 13.0. 2004, SPSS Inc., Chicago. IL) or Microsoft Excel 2010 (Microsoft Cooperation, Redmond, WA). A *P* < 0.05 was set a priori to be statistically significant.

## Results

The mean “Arctic Root” tablet weights for all extractions were 168 ± 9.0 mg and extraction recovery was 43.4 ± 1.7%. CYP2C9 mean enzyme reference (control) activity without inhibitor was 0.378 ± 0.061 pmol metabolite/(pmol enzyme min). The IC_50_ value of the positive control inhibitor sulfaphenazole was 0.25 ± 0.1 *μ*mol/L (Fig. S1).

**Figure 1:**
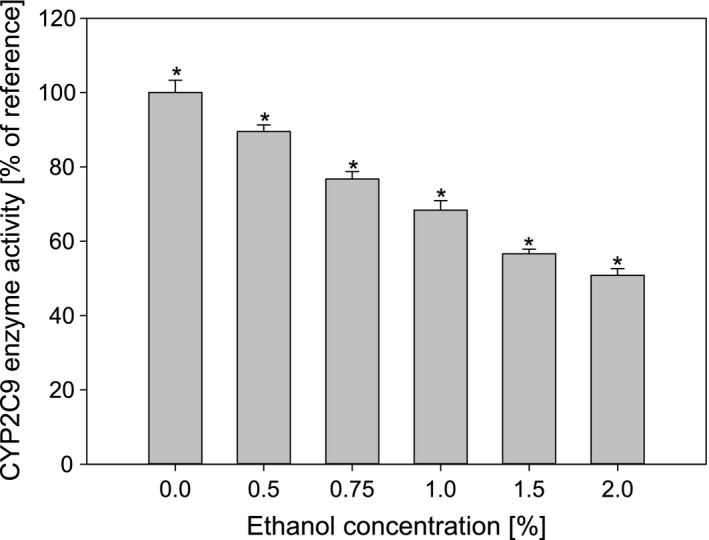
In vitro CYP2C9 enzyme inhibition by increasing ethanol concentrations. Ethanol concentrations ranged from 0.0 to 2.0%. The bars represent mean enzyme activities ± SD (*n* = 3), in the presence of increasing ethanol concentrations. *Two‐tailed *t*‐test between concentrations, *P* < 0.05.

Figure 1 shows the effect of ethanol in relevant in vitro methodological concentrations. At the maximum ethanol concentration tested of 2.0% the CYP2C9 enzyme activity was reduced to 50.8 ± 1.8%.

**Figure 2:**
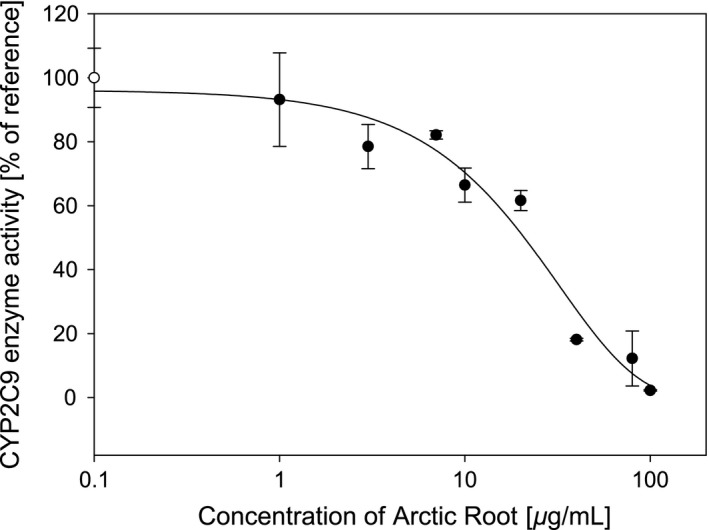
In vitro CYP2C9 enzyme inhibition by ethanol extracts of the commercial *R. rosea* product “Arctic Root”. Herbal extract concentrations ranged from 1 to 100 *μ*g/mL. The filled circles represent mean enzyme activities ± SD (*n* = 3) in the presence of “Arctic Root”. The open circle represent mean enzyme activity (control) ± SD (*n* = 3) without the presence of “Arctic Root”.

Figure 2 shows the inhibition by *R. rosea* “Arctic Root” extract on CYP2C9‐mediated metabolism of losartan. The IC_50_ value was calculated to be 19.2 ± 2.7 *μ*g/mL.

Time‐dependent inhibition was evaluated by preincubating CYP2C9 in the presence of NADPH and “Arctic Root” extract (20 *μ*g/mL), but without losartan for 0, 15, 30, and 45 min. Results were CYP2C9 activities of 49.5 ± 0.0, 53.8 ± 0.1, 54.5 ± 0.1, and 51.2 ± 0.1% compared to reference without inhibitor, respectively. NADPH dependency was evaluated by preincubating CYP2C9 with “Arctic Root” extract (20 *μ*g/mL) with and without NADPH for 45 min. Results were CYP2C9 activities of 51.2 ± 0.1 and 52.2 ± 0.2%, respectively. No significant differences were found (two‐tailed *t*‐test) for time‐dependent or NADPH‐dependent inhibition.

**Figure 3:**
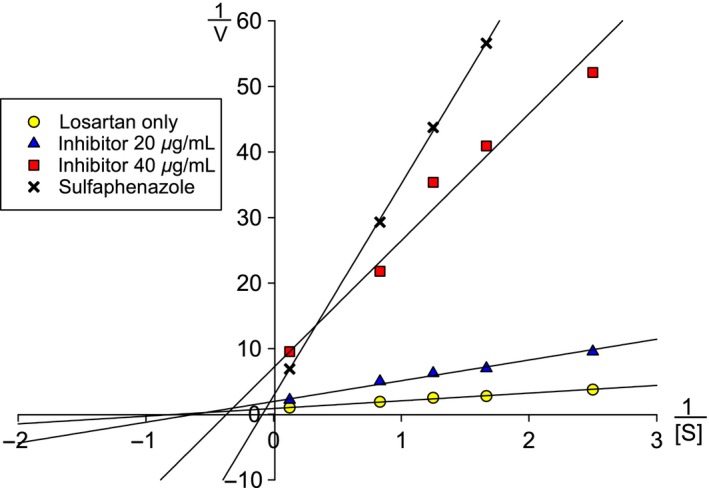
Lineweaver‐Burk inhibition plot on CYP2C9 enzyme activity, using losartan as substrate. Extract of the commercial *R. rosea* product “Arctic Root” as inhibitor is tested at concentrations of 20 and 40 *μ*g/mL. The known competitive inhibitor sulfaphenazole is used as a positive control. All incubations were performed with losartan concentrations of 0.4, 0.6, 0.8, 1.2, and 8 μmol/L. Based on visual inspection of the plot, the effect of “Arctic Root” is consistent with noncompetitive inhibition.

The Lineweaver‐Burk plot is shown in Figure 3. The plot indicates that the commercial *R. rosea* “Arctic Root” is a noncompetitive inhibitor of the CYP2C9‐mediated metabolism of losartan. The corresponding *K*_m_ and *V*_max_ values are given in Table 2. The following equations were used: Substrate only (*y* = 1.1721*x* + 0.8755), sulfaphenazole (*y* = 32.3091*x* + 2.833), inhibitor 20 *μ*g/mL (*y* = 3.1626*x* + 1.9445), and inhibitor 40 *μ*g/mL (*y* = 19.3507*x* + 7.1215). Losartan concentrations for all incubations were 0.4, 0.6, 0.8, 1.2, and 8 *μ*mol/L. *K*_i_ values were also calculated from the Lineweaver‐Burk dataset by using the equations given in Table 1.

**Table 2:** Calculated apparent *K*_m_, *V*_max_, and *K*_i_ based upon the Lineweaver‐Burk plot. Inhibitor is the commercial *R. rosea* product “Arctic Root”

	*K*_m_ (app)	*V*_max_ (app)	*K*_i_
Losartan^a^	1.134 *μ*mol/L	1.142 *μ*mol/L	
Sulfaphenazole^a^	11.405 *μ*mol/L	0.353 *μ*mol/L	0.11 *μ*mol/L
Inhibitor 20 *μ*g/mL^b^	1.626 *μ*g/mL	0.514 *μ*g/mL	16.37 *μ*g/mL
Inhibitor 40 *μ*g/mL^b^	2.717 *μ*g/mL	0.140 *μ*g/mL	5.59 *μ*g/mL

a *K*_m_ and *V*_max_ in the absence of inhibitor.

b *K*_m_ (app) and *V*_max_ (app) are apparent *K*_m_ and *V*_max_ in the presence of inhibitor.

## Discussion

In this study, the in vitro inhibitory potential and inhibition characteristics of the commercial *R. rosea* product “Arctic Root” was investigated using cDNA baculovirus‐expressed human CYP2C9 Supersomes. In all experiments, specific positive inhibition control activities and analytical determination of metabolites fulfilled preset international acceptance criteria (Food and Drug Administration 2001). The *K*_m_ and *V*_max_ values calculated for the substrate losartan in the absence of any inhibitor in our study is similar to previously published data (Maekawa et al. 2009). For the control inhibitor sulfaphenazole, the calculated *K*_i_ falls within the range of similar studies (Brown et al. 2006).

The mean “Arctic Root” tablet weight and extraction recovery corresponded well with previously published results for the same product with identical LOT number. In addition, we have previously verified the product quality by quantification of the established *R. rosea* markers salidroside, tyrosol, rosavin, rosarin, and rosin (Thu et al. 2016b). The IC_50_ value of the positive control sulfaphenazole on CYP2C9 activity was 0.25 ± 0.1 *μ*mol/L, and falls within the range found in studies using similar methodology (Dinger et al. 2014).

The influence of ethanol at relevant methodological concentrations presented in Figure 1 demonstrates a strong decrease in activity with increasing ethanol concentration. This contradicts the findings of Busby et al. (1999) where only a small inhibition of 7 ± 6% at 3% ethanol concentration was found when quantifying the CYP2C9‐mediated formation of 4′‐hydroxydiclofenac from diclofenac. In fact, the influence of ethanol on CYP2C9 activity seems to be substrate dependent, with a significant inhibition on warfarin metabolism at 0.1 vol% and no inhibition of diclofenac metabolism at 1 vol% (Tatsumi et al. 2009). In general, the influence of ethanol on CYP2C9 differs from that on CYP2D6, where Hellum and Nilsen (2007) found a biphasic effect with a small inhibition at 0.1%, a significant activation at 0.5%, 0.8%, 1.1%, 1.5%, and 5%, and a significant inhibition at 8% and 15%. When comparing our observed 32% inhibition of CYP2C9 at 1% ethanol with the investigated CYP enzymes at similar ethanol concentration in the study by Busby et al. (1999), we found that the CYP2C9 inhibition was more potent than for CYP1A2, CYP2A6, CYP2C8, and CYP3A4, and weaker than for CYP1A1, CYP2B6, CYP2C19, and CYP2D6. These findings show that care must be taken when conducting experiments with CYP2C9 where ethanol is used as a solvent, to ensure an identical ethanol concentration throughout the experimental setup.

In this study, we found an in vitro CYP2C9 IC_50_ value of 19.2 ± 2.7 *μ*g/mL. In a previous investigation of the inhibition of six commercial *R. rosea* products including “Arctic Root” on CYP1A2, CYP2D6, and CYP3A4, IC_50_ values of 19.5 ± 5, 30.1 ± 3.6, and 11.6 ± 1.1 *μ*g/mL, respectively, were found (Thu et al. 2016a). This places the in vitro CYP2C9 inhibition potential in line with our previous study. However, CYP2C9 was the only affected enzyme in an in vivo experiment investigating interactions with CYP1A2, CYP2C9, CYP2C19, CYP2D6, and CYP3A4 in man using “Arctic Root” as test compound (Thu et al. 2016b). One would perhaps expect a more potent in vitro CYP2C9 inhibition based on the in vivo results, but the present result illustrates the challenges of predicting possible in vivo interactions by interpretation of in vitro inhibitory data. Similar findings have been demonstrated for milk thistle, where in vitro CYP3A4 inhibition by its constituents silymarin and silibinin produced IC_50_ values ranging from 27 to 60 *μ*mol/L. The constituents were classified as moderate inhibitors, but no inhibition could be reproduced in vivo in a study in humans (Goey et al. 2013). A main challenge of in vivo prediction is the bioavailability of the active constituent responsible for the inhibition, which in many cases remains unknown (Goey et al. 2013). No time‐dependent inhibition or NADPH dependency was found for “Arctic Root” during preincubation, demonstrating that the observed inhibition is not of mechanistic type. A mechanistic inhibition has a more complex clinical effect, where in addition the timing of intake of the inhibitor relative to the substrate intake is an important factor for a possible effect in vivo (Lin and Lu 1998). Our results show that this will not be the case for *R. rosea*.

The commercial *R. rosea* product “Arctic Root” displayed a noncompetitive inhibition by visual interpretation of the Lineweaver‐Burk plot given in Figure 3. Similar studies have also found a noncompetitive inhibition of CYP2C9 by other herbal products like *Bacopa monnieri* (IC_50_/*K*_i_ = 36.49/12.5 *μ*g/mL) (Ramasamy et al. 2014), *Hochuekki‐to*, and *Sairei‐to* (*K*_i_ of 0.7–0.8 mg/mL and 0.25 mg/mL, respectively) (Takahashi et al. 2003). The *Bacopa monnieri* inhibition is in line with our *R. rosea* inhibition, but *Hochuekki‐to* and *Sairei‐to* are significantly weaker. We could not find any published in vivo studies for these herbs. Although our interpretation of the overall results from the Lineweaver‐Burk plots indicates a noncompetitive inhibition, it is also possible to argue a dual inhibition mode with noncompetitive inhibition at low concentrations (20 *μ*g/mL) and competitive at high concentration (40 *μ*g/mL). This shift is uncommon, but similar findings have previously been described for gentiopicroside on CYP2A6 (Deng et al. 2013), amiodarone on triiodothyronine binding to thyroid hormone receptor beta 1 (Drvota et al. 1995), and metyrapone on *N*‐demethylation of aminopyrine by mixed function oxidase cytochrome P‐450 in rat (Roots and Hildebrandt 1973). A noncompetitive inhibition should also give a *K*_i_ value close to the IC_50_ value for any inhibitor concentration. The calculated *K*_i_ for the 40 *μ*g/mL inhibitor concentration was somewhat lower. One explanation for a shift like this, if there is any, could be linked to different constituents exerting effects toward the enzyme at different herbal concentrations. A competitive inhibitor has a *K*_i_ value of approximately half the corresponding IC_50_ value. In our study, sulfaphenazole, a known competitive inhibitor, had a *K*_i_ of 0.11 *μ*mol/L and a IC_50_ of 0.25 *μ*mol/L.

In the interpretation of both the in vitro and in vivo inhibitory potential and characteristics of *R. rosea* on CYP enzyme activity, the potential differences in biochemical activity of multiple unknown constituents must also be taken into consideration. The supposed bioactive constituents of *R. rosea* are salidroside, tyrosol, rosavin, rosarin, and rosin (Ming et al. 2005; Elameen et al. 2010). Approximately 140 different constituents are identified in *R. rosea* (Panossian et al. 2010), but the constituents responsible for the observed inhibition is yet to be identified. Hellum et al. (2010) examined whether the concentration of the supposed bioactive constituents could be correlated with CYP3A4 enzyme inhibition, but with no significant findings. Moreover, no correlation between the supposed bioactive constituents and in vitro CYP1A2, CYP2D6, and CYP3A4 enzyme activities was found in a previous publication from our group (Thu et al. 2016a). However, a moderate noncompetitive in vitro CYP2D6 inhibition by the relatively unknown *R. rosea* constituents rhodiosin and rhodionin has recently been published with IC_50_ values of 0.47 and 0.19 *μ*g/mL, respectively (Xu et al. 2013). For better prediction of the potential in vivo inhibition of commercial *R. rosea* products, the specific constituents responsible for the inhibition must be identified.

In conclusion, this study found a noncompetitive inhibition of CYP2C9 by the commercial *R. rosea* product “Arctic Root” and adds to the increasing evidence of both in vitro and in vivo CYP inhibitory potential of *R. rosea*. Further studies should evaluate possible CYP enzyme effects of other commercially available *R. rosea* products and in more detail attempt to identify which of the *R. rosea* constituents that are responsible for these effects.

## Disclosure

The authors declare that they have no conflict of interest.

## Supporting information


**Figure S1.** In vitro CYP2C9 enzyme inhibition by the positive control inhibitor sulfaphenazole.
