# Supplementary material for: Noncompetitive inhibition of human CYP2C9 in vitro by a commercial Rhodiola rosea product
Source: Pharmacol Res Perspect. 2017 Jun 5;5(4):e00324. doi: 10.1002/prp2.324 (PMC5684854; doi:10.1002/prp2.324)
Supplement: Supplementary file 2 [file PRP2-5-e00324-s002.docx]

**SUPPLEMENTARY MATERIAL**


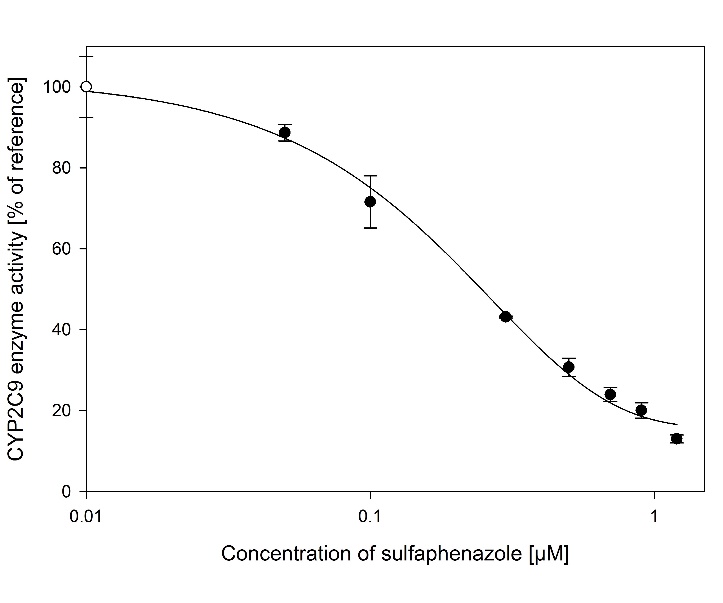


**SFig. S1 *In vitro* CYP2C9 enzyme inhibition by the positive control inhibitor sulfaphenazole.** Sulfaphenazole concentrations ranged from 0.01 – 1.2 µM. The filled circles represent mean enzyme activities ± SD (n = 3), in the presence of sulfaphenazole. The open circle represent mean enzyme activity (control) ± SD (n = 3), without the presence of sulfaphenazole.

**LC-MS/MS measurements**

In short: LC-MS/MS: Shimadzu LC20AD LC system (Shimadzu Scientific Instruments, Columbia, SC, USA), coupled with an ABSciex 5500 triple quad mass spectrometer (ABSciex, Concord, ON, Canada). Column: Waters Xbridge C18 column (21 x 100 mm, 3.5 µm) (Waters, Palo Alto, CA, USA). Mobile phase: water w/0.1% formic acid (A) and acetonitrile w/0.1% formic acid (B) with gradient 0 – 1 min 15% B, 1 – 4 min B increased to 80%, 4 – 7 min 80% B, 7 – 7.5 min B decreased to 15%, 7.5 – 9 min 15% B. Flow rate: 0.3 ml/min, total run time 9 min. Injection volume 5 µl. Autosampler and column oven were operated at 10ºC and 30ºC, respectively. The MS parameters were as follows: curtain gas 16 psi, collision gas (CAD) 8 psi, Ion Spray Voltage (IS) 5500 KV, temperature 575 ºC, Ion Source Gas 1 (GS1) 60 psi, Ion Source Gas 2 (GS2) 50 psi. The mass spectrometer was operated in positive mode with electrospray ionization and multiple reaction monitoring (MRM). MS settings for each analyte and the internal standard caffeine are given in Supplementary table 1 and 2.

# **STable 1.** Overview of parameters for the mass spectrometrer settings for each of the analytes

| Analyte | Q1 / Q3 | Dwell time | DP | EP | CE | CXP |
| --- | --- | --- | --- | --- | --- | --- |
| Caffeine (IS) | 195.0 / 138.3 | 100 | 40 | 10 | 27 | 12 |
| Losartan | 423.2 / 207.2 | 100 | 101 | 10 | 31 | 12 |
| EXP-3174 | 437.2 / 235.0 | 100 | 111 | 10 | 25 | 14 |

IS = internal standard

Q1 / Q3 = quadrupole 1 / quadrupole 3

Dwell time = time system searches for each mass, each cycle

DP = declustering potential

EP = entrance potential

CE = collision energy

CXP = collision cell exit potential

**STable 2.** Limit of quantitation, linear range of the calibration curve and inter-day precision and accuracy for each of the analytes (n = 6)

| Analyte | LOQ (ng/ml) | Linear range  (ng/ml) | Precision  (% CV) | Accuracy  (%) |
| --- | --- | --- | --- | --- |
| Caffeine (IS) | 5.0 | 5.0 - 500 | 4.6 – 9.6 | 93.0 – 97.3 |
| Losartan | 2.5 | 2.5 - 40 | 4.8 – 11.9 | 89.9 – 97.4 |
| EXP-3174 | 2.5 | 2.5 – 40 | 5.3 – 13.6 | 91.0 – 102.6 |

LOQ = limit of quantitation; CV = coefficient of variation; IS = internal standard
